# Supplementary material for: A biomimetic neural encoder for spiking neural network
Source: Nat Commun. 2021 Apr 9;12:2143. doi: 10.1038/s41467-021-22332-8 (PMC8035177; doi:10.1038/s41467-021-22332-8)
Supplement: Supplementary file 1 — Supplementary Information [file 41467_2021_22332_MOESM1_ESM.pdf]

Supplementary Information

A Biomimetic Neural Encoder for Spiking Neural Network

Shiva Subbulakshmi Radhakrishnan[1], Amritanand Sebastian[1], Aaryan Oberoi[1], Sarbashis Das[2], and Saptarshi Das[1,3,4,]*

[1]Department of Engineering Science and Mechanics, Pennsylvania State University, University Park, PA 16802, USA

[2]Department of Electrical Engineering, Pennsylvania State University, University Park, PA 16802, USA

[3]Department of Materials Science and Engineering, Pennsylvania State University, University Park, PA 16802, USA

[4]Materials Research Institute, Pennsylvania State University, University Park, PA 16802, USA

Supplementary Figure 1

a) Schematic of the neural encoder with the top-gate as the stochastic sampling terminal and the back-gate as the presynaptic voltage terminal. b) Corresponding transfer function i.e. I_{PSC} *versus* V_{PSV} measured at a drain bias, V_{DS} = 1 V, for different top gate voltages (V_{TG}). c) Schematic of the neural encoder with the back-gate as the stochastic sampling terminal and the top-gate as the presynaptic voltage terminal. d) Corresponding transfer function i.e. I_{PSC} *versus* V_{PSV} measured at a drain bias, V_{DS} = 1 V, for different back-gate voltages (V_{TG}). Note that the dual-gated MoS_2 field effect transistor (FET) allows interchangeable presynaptic terminal and encoding terminal based on the application requirements. For example, different analog inputs can be encoded simultaneously if the top-gate is used as the presynaptic terminal in a chip containing multiple neural encoders with the global back-gate acting as the common stochastic sampling terminal. Similarly, the same analog input applied to the presynaptic back-gate terminal can be encoded into different spike trains by adjusting the transfer function of individual neural encoders through the top-gate sampling terminal.

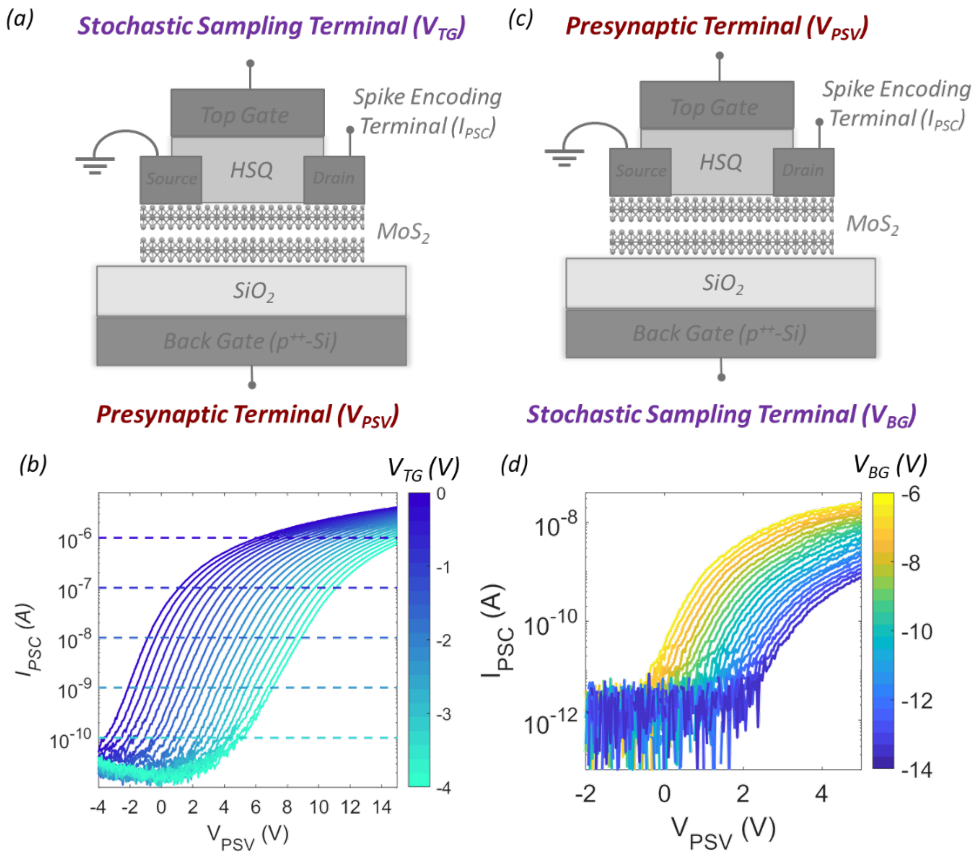

Supplementary Note 1

Note that the on-off current ratio can be orders of magnitude lower for the top-gate voltage sweep (V_{TG}) compared to the back-gate voltage sweep (V_{BG}) as seen in ***Supplementary Figure 1b and 1d***. In fact, the on-off ratio for V_{TG} sweep depends on V_{BG}. This is because of the fact that in a top-gated geometry, MoS_2 channel underneath the contacts cannot be gated by the top-gate, whereas the global back-gate can control the entire MoS_2 channel including the extension regions underneath the contacts [1, 2]. As such, the top-gate characteristics can be severely limited by the channel resistance underneath the contacts. For instance, as the V_{BG} becomes more negative both the channel and the channel underneath the contacts are biased in the subthreshold regime, where the resistance of MoS_2 increases exponentially. While the channel can be switched to the on-state by applying positive V_{TG} values, the extensions underneath the contacts remain unaltered adding extra resistance that dominates the overall current transport. Since this extension resistance due to the MoS_2 channel underneath the contacts depend on V_{BG} so is the on-off ratio obtained during the V_{TG} sweep. Note that this phenomenon is a direct consequence of the fact that unlike Si, MoS_2 FETs lack degenerate doping underneath the contacts. Several doping strategies are being developed to circumvent these challenges, which are beyond the scope of discussion in the context of the present work [3].

Supplementary Figure 2

Complete neuromorphic circuit used to obtain neural encoding for different LED illumination levels.

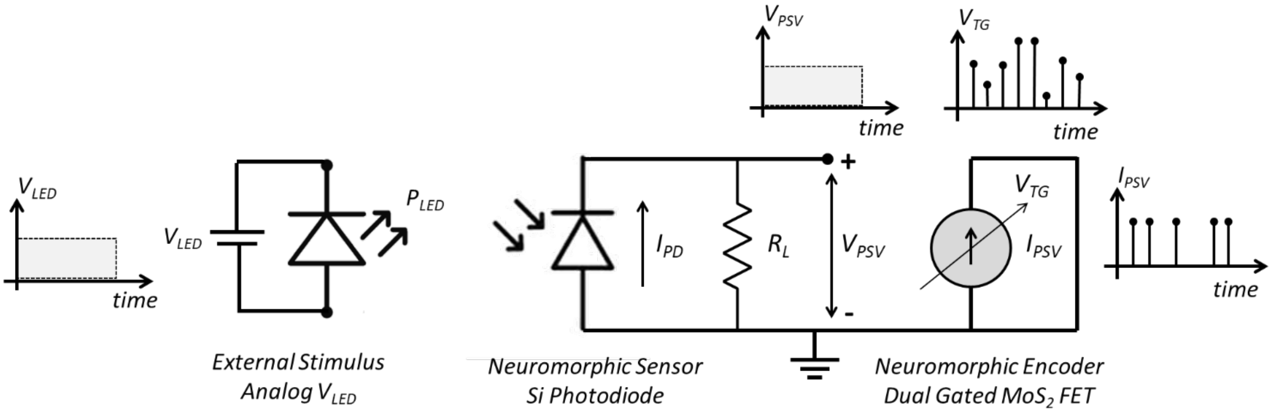

Supplementary Figure 3

Distribution (i.e. number of occurrences) of interspike interval corresponding to different presynaptic voltages (V_{PSV}) for rate-based encoding using our biomimetic encoder, when the magnitude of V_{TG} pulses are randomly sampled from a Gaussian distribution with mean, μ_{TG} = -2.5 V, and standard deviation σ_{TG} = 0.8 V.

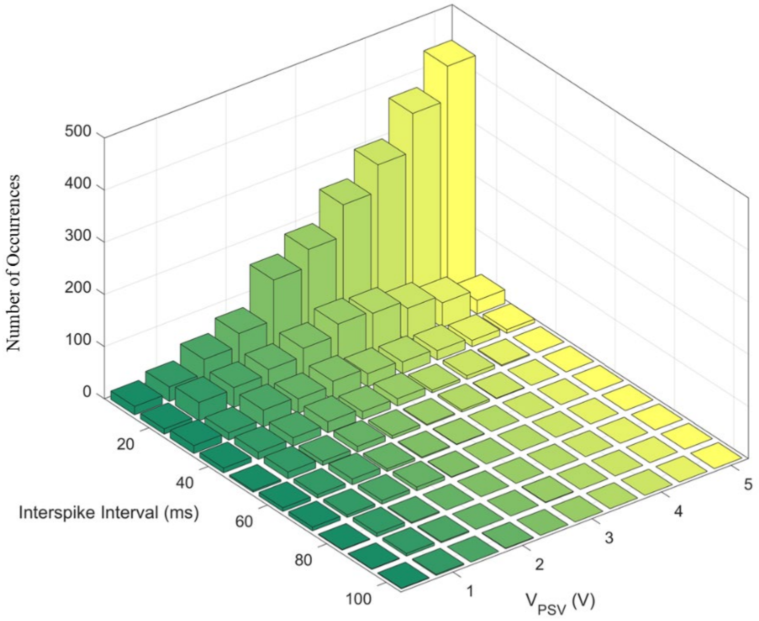

Supplementary Figure 4

Total spike counts corresponding to different presynaptic voltages (V_{PSV}) and for different σ_{TG} for spike count-based encoding.

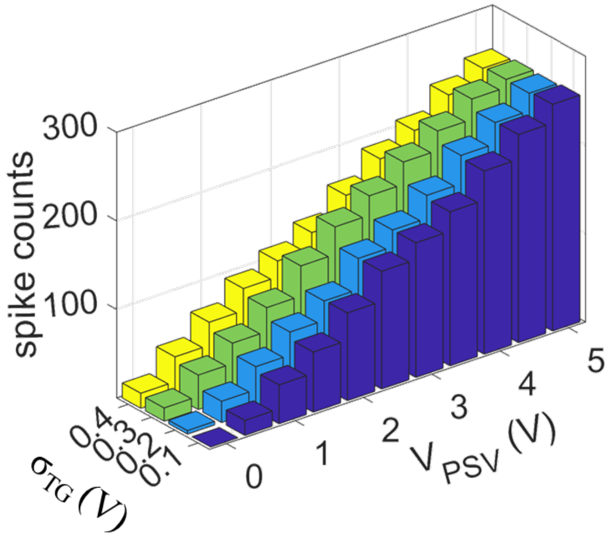

Supplementary Figure 5

Distribution (i.e. number of occurrences) for the spike timing corresponding to different presynaptic voltages (V_{PSV}) for σ_{TG} = 0.1 V across 16 trials for spike timing-based encoding.

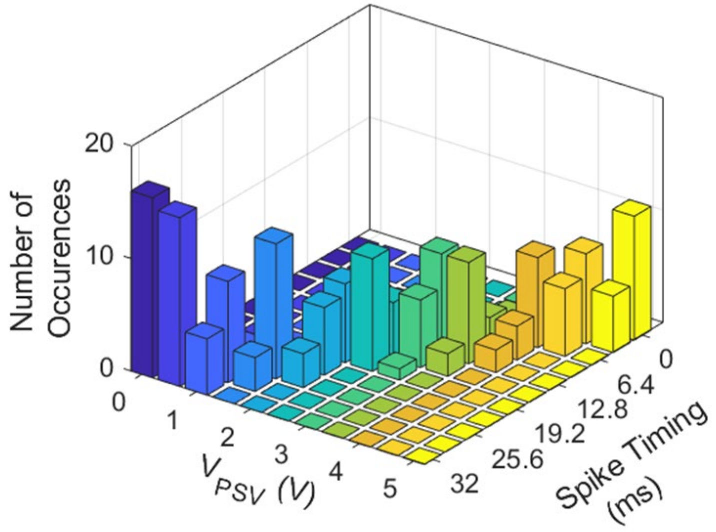

Supplementary Note 2

Benchmarking of our neural encoder with other types of spike encoders

Reference	*Technology*	*Hardware Encoding*	*Spike Encoding Type*			*Energy per synaptic event*
			Rate	*Count*	*Temporal*	
[4]	Memristor	☑	☒	☒	☑	25 pJ
[5]	CMOS 180nm	☒	☑	☒	☑	225 fJ
[6]	CMOS 65nm	☑	☑	☒	☒	40 fJ
[7]	MTJ	☒	☑	☒	☒	48 fJ
[8]	PCM	☒	☑	☒	☒	-
[9]	FPGA	☑	☒	☒	☑	21 nJ
[10]	CBRAM	☒	☒	☒	☑	-
This work	2D FET	☑	☑	☑	☑	< 3 pJ

Supplementary Figure 6

Time evolution of correlation coefficient (CC) between the original and the encoded Cameraman image using various neural encoding algorithms.

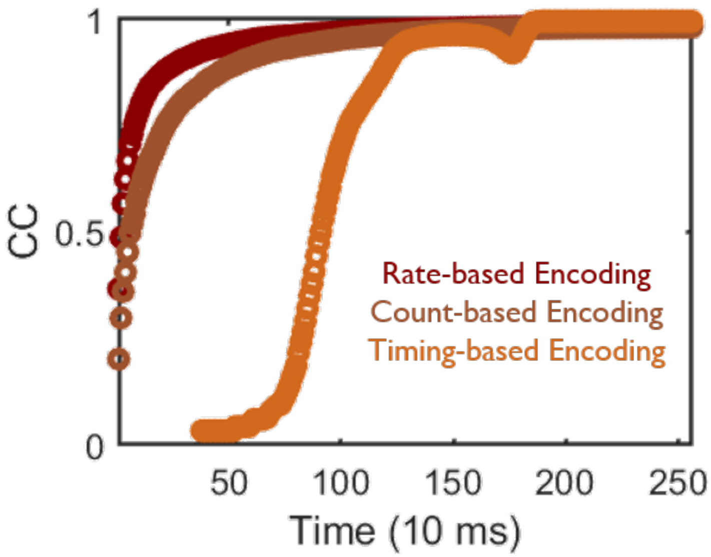

Supplementary Figure 7

Dual sweep a) back-gate and b) top-gate transfer characteristics of dual-gated multilayer MoS_2 FET.

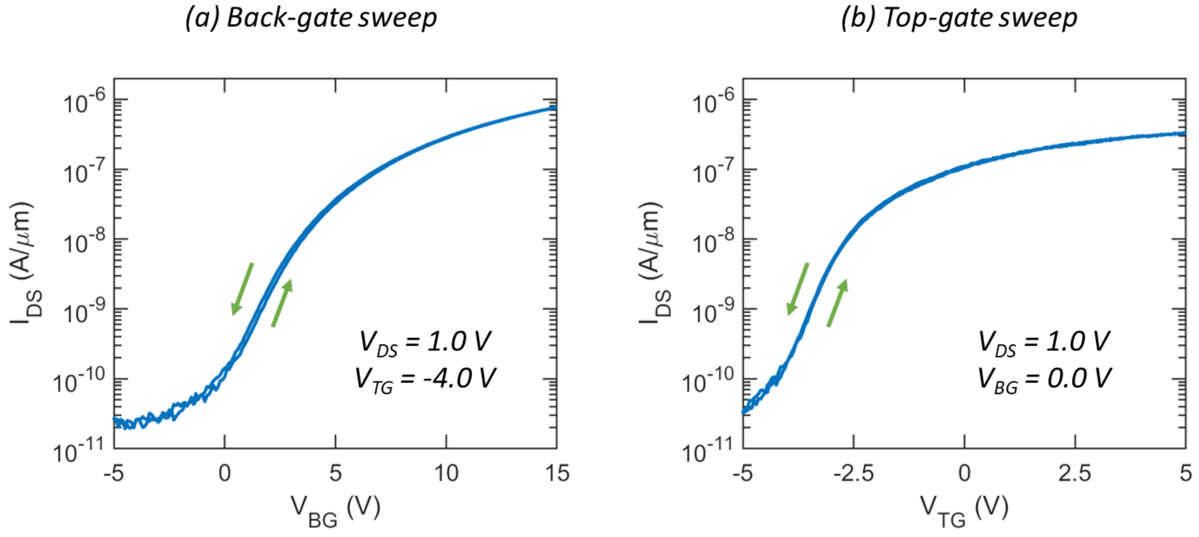

References

[1] J. R. Nasr and S. Das, "Seamless Fabrication and Threshold Engineering in Monolayer MoS2 Dual-Gated Transistors via Hydrogen Silsesquioxane," *Advanced Electronic Materials,* vol. 5, p. 1800888, 2019.

[2] J. R. Nasr, D. S. Schulman, A. Sebastian, M. W. Horn, and S. Das, "Mobility Deception in Nanoscale Transistors: An Untold Contact Story," *Advanced Materials,* vol. 31, p. 1806020, 2019.

[3] A. J. Arnold, D. S. Schulman, and S. Das, "Thickness Trends of Electron and Hole Conduction and Contact Carrier Injection in Surface Charge Transfer Doped 2D Field Effect Transistors," *ACS Nano,* vol. 14, pp. 13557-13568, 2020/10/27 2020.

[4] I. Gupta, A. Serb, A. Khiat, R. Zeitler, S. Vassanelli, and T. Prodromakis, "Real-time encoding and compression of neuronal spikes by metal-oxide memristors," *Nat Commun,* vol. 7, p. 12805, Sep 26 2016.

[5] C. Zhao, B. T. Wysocki, Y. Liu, C. D. Thiem, N. R. McDonald, and Y. Yi, "Spike-Time-Dependent Encoding for Neuromorphic Processors," *ACM Journal on Emerging Technologies in Computing Systems,* vol. 12, pp. 1-21, 2015.

[6] I. Sourikopoulos, S. Hedayat, C. Loyez, F. Danneville, V. Hoel, E. Mercier*, et al.*, "A 4-fJ/Spike Artificial Neuron in 65 nm CMOS Technology," *Front Neurosci,* vol. 11, p. 123, 2017.

[7] A. Sengupta, P. Panda, P. Wijesinghe, Y. Kim, and K. Roy, "Magnetic Tunnel Junction Mimics Stochastic Cortical Spiking Neurons," *Sci Rep,* vol. 6, p. 30039, Jul 21 2016.

[8] S. Ambrogio, N. Ciocchini, M. Laudato, V. Milo, A. Pirovano, P. Fantini*, et al.*, "Unsupervised Learning by Spike Timing Dependent Plasticity in Phase Change Memory (PCM) Synapses," *Front Neurosci,* vol. 10, p. 56, 2016.

[9] A. J. Hill, J. W. Donaldson, F. H. Rothganger, C. M. Vineyard, D. R. Follett, P. L. Follett*, et al.*, "A Spike-Timing Neuromorphic Architecture," presented at the 2017 IEEE International Conference on Rebooting Computing (ICRC), 2017.

[10] M. Suri, D. Querlioz, O. Bichler, G. Palma, E. Vianello, D. Vuillaume*, et al.*, "Bio-Inspired Stochastic Computing Using Binary CBRAM Synapses," *IEEE Transactions on Electron Devices,* vol. 60, pp. 2402-2409, 2013.
