# Supplementary material for: A biomimetic neural encoder for spiking neural network
Source: Nat Commun. 2021 Apr 9;12:2143. doi: 10.1038/s41467-021-22332-8 (PMC8035177; doi:10.1038/s41467-021-22332-8)
Supplement: Supplementary file 3 — Description of Additional Supplementary Files [file 41467_2021_22332_MOESM3_ESM.pdf]

Description of Additional Supplementary Files

Supplementary Movie 1 for real time encoding of different LED intensities into stochastic spike trains.

Supplementary Movie 2 show the time evolution of encoded images over time for rate-based, count-based, and timing-based encoding.
